# Minimally Invasive Versus Full Sternotomy Approaches in Mitral Valve Surgery for Infective Endocarditis: A Retrospective Comparative Analysis

**DOI:** 10.3390/diseases13050135

**Published:** 2025-04-28

**Authors:** Elisa Mikus, Mariafrancesca Fiorentino, Diego Sangiorgi, Antonino Costantino, Simone Calvi, Elena Tenti, Elena Tremoli, Alberto Tripodi, Carlo Savini

**Affiliations:** 1 Cardiovascular Department, Maria Cecilia Hospital, GVM Care & Research, 48033 Cotignola, RA, Italy; elisamikus@yahoo.it (E.M.); francescafiorentino@hotmail.it (M.F.); antonino@costantinorc.com (A.C.); scalvi@gvmnet.it (S.C.); etenti@gvmnet.it (E.T.); etremoli@gvmnet.it (E.T.); albertotripodi@hotmail.com (A.T.); csavini@gvmnet.it (C.S.); 2 Department of Experimental Diagnostic and Surgical Medicine (DIMEC), University of Bologna, 40126 Bologna, BO, Italy

**Keywords:** endocarditis, minimally invasive, mitral valve surgery, aortic valve replacement

## Abstract

**Background:** This study evaluates the outcomes of isolated mitral valve surgery for infective endocarditis performed via conventional full sternotomy or minimally invasive right minithoracotomy. While minimally invasive surgery (MIS) is well-established for elective mitral procedures, its role in infective endocarditis remains less explored due to the complexity of the disease. **Methods:** A retrospective analysis of 134 patients who underwent isolated mitral valve surgery for infective endocarditis between January 2010 and March 2024 was conducted. Patients were divided into two groups based on the surgical approach: full sternotomy (n = 94) and MIS via right minithoracotomy (n = 40). Variables analyzed included preoperative characteristics, intraoperative details, and postoperative outcomes, such as mortality, complications, and hospital stay duration. Given significant baseline differences, inverse probability weighting was applied for comparability. **Results:** Mitral valve replacement was performed in 77% of cases. After adjustment, the MIS group demonstrated shorter intensive care unit stays (*p* = 0.018), with no significant differences in in-hospital mortality (*p* = 0.145) or total hospitalization length (*p* = 0.151). **Conclusions:** Minimally invasive mitral valve surgery is a safe and effective alternative to sternotomy in infective endocarditis, offering comparable outcomes with shorter ICU stays. Further research is needed to refine patient selection and validate these findings.

## 1. Introduction

Infective endocarditis (IE) is a severe condition affecting the heart valves and endocardium, leading to high rates of morbidity and mortality [1]. The mitral valve is the most frequently involved, followed by the aortic, tricuspid, and pulmonary valves, often with extension to nearby cardiac structures. Over the past years, minimally invasive surgical techniques have gained widespread acceptance as the preferred approach for isolated mitral valve disease in many cardiac surgery centers worldwide. Compared to conventional full sternotomy, this approach has demonstrated comparable safety and efficacy, while offering additional benefits such as reduced postoperative pain, decreased transfusion requirements, shorter hospital stays, faster recovery, improved cosmetic outcomes, and lower reliance on rehabilitation services after discharge [2,3,4].

Continuous advancements in surgical techniques and increasing operator experience have gradually extended the indications for minimally invasive cardiac surgery, including its use in more complex and high-risk scenarios such as IE. Nonetheless, current clinical guidelines remain cautious. Specifically, the latest European Society of Cardiology (ESC) guidelines do not provide specific recommendations regarding the use of minimally invasive surgery for IE, highlighting the need for further data in this setting [5].

Although a few studies have explored the application of minimally invasive techniques in the context of IE, most are descriptive in nature or compare outcomes between endocarditis and non-endocarditis etiologies within minimally invasive cohorts [6,7,8,9,10,11]. To our knowledge, only limited evidence directly compares the outcomes of minimally invasive versus conventional surgery in patients undergoing mitral valve procedures specifically for IE [12,13].

The present study seeks to address this gap. We provide a comparative analysis of minimally invasive versus sternotomy approaches in a cohort of patients with mitral valve endocarditis. A key strength of our study lies in the use of inverse probability of treatment weighting (IPTW), which allowed us to balance both preoperative clinical variables and echocardiographic characteristics between groups. This methodology minimizes selection bias and enhances the reliability of outcome comparisons. As such, we believe this study offers valuable new evidence supporting the feasibility and safety of minimally invasive mitral valve surgery in the treatment of IE, contributing meaningful data to guide surgical decision-making in this complex patient population.

## 2. Materials and Methods

Demographic and essential baseline parameters, including age, gender, body mass index, renal function (creatinine clearance), preoperative clinical condition, cardiovascular risk profile, functional status, left ventricular ejection fraction, and EuroSCORE II, were systematically gathered and documented. Furthermore, intraoperative variables and early postoperative outcomes were meticulously recorded. Detailed echocardiographic assessments were performed both preoperatively and postoperatively to evaluate cardiac performance and the effectiveness of the intervention.

### 2.1. Study Design and Outcomes

Between January 2010 and March 2024, a total of 633 patients underwent surgical treatment for infective endocarditis at our institution. This single-center retrospective study specifically examines 134 patients who underwent isolated mitral valve surgery. Patients were classified based on the surgical approach, comparing conventional full sternotomy to minimally invasive techniques, including right thoracotomy with either direct vision or endoscopic-assisted mitral valve surgery. While no formal power calculation was performed, all eligible patients were included in the analysis.

The study protocol was approved by the Romagna Ethics Committee on 30 June 2023 (protocol no. 4497/2023 I.5/95). Due to the retrospective nature of the study, the requirement for individual informed consent was waived. Data were retrieved from medical records and systematically entered into a dedicated registry, with extensive efforts undertaken to minimize missing data. Missing values were assumed to be missing completely at random, arising from documentation inconsistencies in the original clinical records. Consequently, only complete cases were considered for the final analysis.

Patients were stratified according to the surgical approach: median sternotomy (n = 94) or minimally invasive techniques (n = 40).

### 2.2. Statistical Analysis

After checking for normal distribution using the Shapiro-Wilk test, continuous variables were reported as median and interquartile range (IQR) and compared with the Mann-Whitney test; categorical variables were reported as absolute number and frequencies and compared with the chi-squared test or Fisher’s exact test as appropriate. In order to reduce selection bias, inverse probability of treatment weighting (IPTW) with the covariate balancing propensity score (CBPS) method was applied; baseline and echo characteristics were used to build the weights used for all weighted analysis; weights were truncated according to the common support; absolute standardized mean differences (ASMD) were reported in order to assess balancing across groups; variables with ASMD < 0.2 were considered as balanced. Covariates that were still unbalanced after IPTW (PAPs > 50 mmHg) were included in all models in order to correct for residual imbalance. Missing data imputation was performed using Random Forest.

Weighted logistic regression models and generalized linear models (GLM) with Gamma distribution and log link function were used to assess differences in binary or continuous in-hospital complications, respectively; deviance residuals were analyzed for normality.

Weighted Kaplan-Meier curves and a weighted Log-Rank test for the risk of IE recurrence were reported at a two-year follow-up.

All analyses were performed with R 4.4.0 (R Foundation for Statistical Computing, Vienna, Austria); *p*-values < 0.05 were considered statistically significant.

## 3. Results

### 3.1. Study Population

The baseline characteristics of the patient population, divided into two groups based on the surgical approach, are summarized in Table 1. The study included 134 patients diagnosed with infective endocarditis involving the mitral valve. The cohort had a median age of 66 years (IQR: 54–73), with 53.0% of patients being male. Patients were categorized by surgical technique: median sternotomy (n = 94) and minimally invasive approach through a right minithoracotomy (n = 40).

Significant differences were observed between the two groups. Specifically, patients treated with a minimally invasive approach had lower logistic EuroSCOREs (*p* = 0.002), were less likely to have an abscess (*p* = 0.022), and were predominantly affected by Streptococcus infections (*p* = 0.023). Moreover, non-aureus Staphylococcus infections were less frequent in the minimally invasive surgery group compared to the full sternotomy group (*p* = 0.021).

Among patients undergoing minimally invasive surgery, the majority had endocarditis on a native valve rather than a prosthetic valve (*p* = 0.032). Additionally, the proportion of patients with prior surgical interventions—whether on a cardiac valve, coronary revascularization, or other procedures—was significantly lower (*p* = 0.003).

To mitigate selection bias, IPTW was applied, resulting in a balanced cohort for analysis (Table 1).

**Figure 1:**
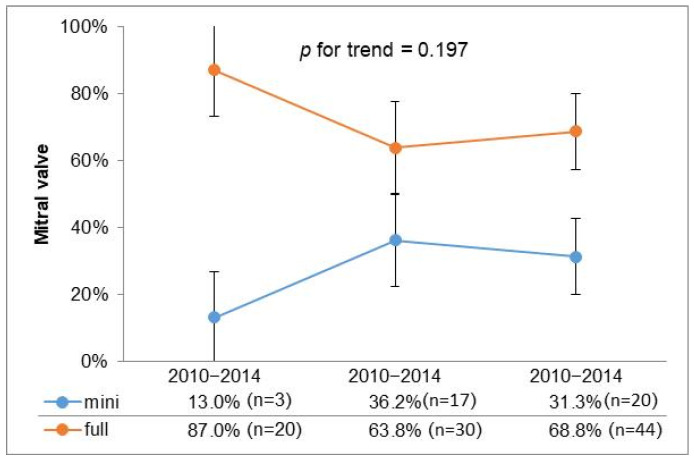
Temporal trend for surgical access.

To further investigate the utilization of minimally invasive surgery over time, we stratified the past 15 years into three consecutive five-year intervals; the graphical representation illustrates a stable trend in the adoption of minimally invasive approaches in recent years (Figure 1).

### 3.2. Postoperative Outcomes and Mortality

Surgical data, adjusted using IPTW, are presented in Table 2, while postoperative outcomes for the study population are detailed in Table 3.

Regarding valve replacement, in the minimally invasive group, 32 patients received a biological prosthesis and 6 received a mechanical one, whereas in the median sternotomy group, 42 patients received a biological prosthesis and 39 a mechanical one. Mitral valve repair was performed in 45% of patients in the minimally invasive group and in 14% of patients in the sternotomy group.

After IPTW, the full sternotomy group exhibited worse outcomes, including prolonged intensive care unit (ICU) stays (*p* = 0.018), higher incidence of pacemaker implantation (*p* = 0.014), stroke (*p* = 0.009), acute kidney injury (*p* = 0.043), and reoperation for bleeding (*p* = 0.036). No significant difference was detected in terms of median hospital stay (*p* = 0.151) and in-hospital mortality (*p* = 0.145), although patients who underwent minimally invasive surgery exhibited a lower mortality rate.

**Figure 2:**
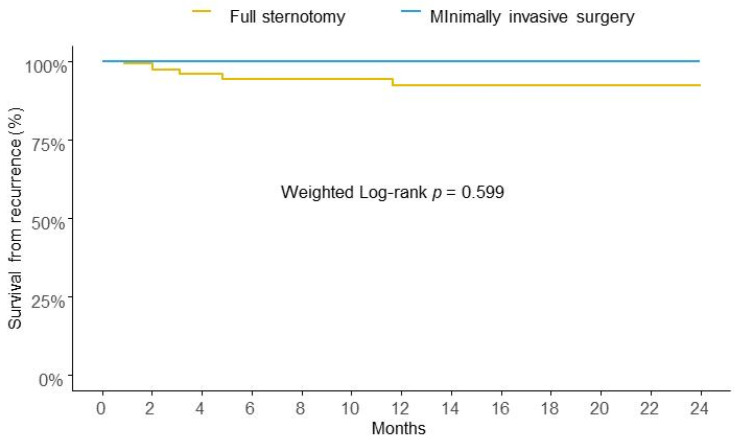
Weighted Kaplan-Meier survival curves for risk of recurrence at two years.

After IPTW, the risk of IE recurrence at two years of follow-up was similar between the two groups in analysis (Figure 2).

## 4. Discussion

Minimally invasive surgery has become the preferred approach for the treatment of mitral valve disease in many centers [14,15], encompassing direct vision techniques, endoscopic assistance, and robotic-assisted procedures. This paradigm shift is driven by numerous advantages as reduced postoperative pain, shorter hospital stays, faster recovery, and improved cosmetic outcomes. Additionally, it is associated with lower rates of transfusion and infection, contributing to enhanced patient satisfaction and quality of life [3,4,14,15]. The success of MIMVS is highly dependent on the experience of the surgical team and the volume of procedures performed. Studies have shown that outcomes improve significantly in centers that specialize in minimally invasive cardiac surgery, where the surgical team is adept at handling complex cases and managing potential complications [16,17,18]. This expertise reduces operative times and enhances procedural safety.

However, in cases of mitral valve disease due to endocarditis, this approach is not as widely accepted, despite reports in the literature supporting its feasibility [19]. Our retrospective study, spanning 15 years, documents the experience of a single center and highlights the evolution of MIMVS utilization, especially from the second five-year period onwards, as shown in Figure 1. A stable trend in the adoption of minimally invasive approaches in recent years suggests a consistent preference for minimally invasive techniques across different types of valvular disease, reflecting advancements in surgical expertise, improved patient selection, and potentially better perioperative outcomes associated with a minimally invasive approach.

Although the study is limited to a small patient cohort, the findings demonstrate positive outcomes in favor of MIMVS. While the mortality rate did not show statistically significant improvements (though there was a positive trend and both approaches report a mortality rate consistent with those reported in the literature) [19], MIMVS exhibited several advantages, including shorter ICU stays and reduced incidences of renal failure, stroke, and sepsis. The overall valve repair rate was 31 out of 34 cases (23%), aligning with literature data [20]. Interestingly, the repair rate was higher in patients treated with MIMVS (18 out of 40; 45%) compared to those treated with traditional sternotomy (13 out of 94; 13.8%). However, this difference was not statistically significant (*p* = 0.624). Mitral valve replacement remains the most common surgical intervention for mitral valve infective endocarditis, with approximately 20–30% of cases of repair, as reported by national registries and consistent with our findings [12,21,22,23,24]. Consistent with Kofler’s findings in 2021 [13], our population also experienced shorter operative times (Table 2), although the difference was not statistically significant.

In the literature, there are few investigations that directly compare MICS to conventional sternotomy in infective endocarditis, but with encouraging results [6,7,8,9]. Many authors agreed that a minimally invasive approach should not be selected in patients with multiple valvular involvement or extensive involvement in non-valvular structures [10,11]. Specifically, we chose to include only patients with isolated mitral valve involvement, as those with multivalvular disease or extensive perivalvular extension often require more complex surgical procedures, such as double or triple valve interventions, or even the Commando procedure. By focusing on patients with an indication for isolated mitral valve surgery due to infective endocarditis, we aimed to establish a homogeneous cohort in which a direct and meaningful comparison between minimally invasive and conventional surgical approaches could be made. This selection ensures that both strategies are technically applicable and allows for a more accurate assessment of outcomes without confounding from procedural complexity. Our experience aligns with that of other isolated centers and supports the recent publication by Franz et al. [7], which asserts that endocarditis is not a contraindication for MIMVS. Instead, it is both feasible and safe, especially in centers with a high degree of experience in minimally invasive mitral valve (MV) surgery. The observed trend over the years, with the consolidation of MIMVS, further corroborates this assertion.

### Limitations

This investigation presents several critical limitations that should be acknowledged when interpreting the results. First, the lack of a randomized design and the retrospective nature of data collection could introduce biases related to patient selection and data accuracy. Additionally, the sample size is limited. The choice between a minimally invasive approach and median sternotomy was at the discretion of the surgeon, influenced by their expertise, preferences, and clinical judgment, rather than through randomization. This factor may have affected the comparability between the treatment groups. Furthermore, disparities in the baseline characteristics of the patient populations, such as preoperative condition and the severity of the disease, could impact the outcomes and limit their applicability to other settings. The study also reflects the practices and outcomes of a single surgical center, which may not be generalizable to other institutions. Lastly, the lack of a formal sample size calculation reduces the statistical power of the results and raises concerns about the reliability and reproducibility of the findings. These constraints underscore the necessity for future multicenter, randomized, and well-powered studies to confirm and build upon these results.

## 5. Conclusions

The findings of this study contribute to the growing body of evidence supporting the feasibility and safety of MIMVS in patients with mitral valve disease due to endocarditis. While the sample size is limited and statistical significance was not achieved in some parameters, the positive trends in clinical outcomes, including reduced ICU stays and lower incidences of complications, underscore the potential benefits of this approach. The increased repair rate with MIMVS compared to traditional sternotomy, although not statistically significant, is noteworthy. Our experience, in line with other reports in the literature, suggests that MIMVS can be a viable and safe option in managing mitral valve endocarditis and should be considered in appropriate clinical settings.

## Figures and Tables

**Table 1:** Pre-operative and baseline characteristics.

	Before IPTW				After IPTW		
	Full	Mini	*p*-Value	SMD	Full	Mini	SMD
n	94	40			170.57	149.59	
Age, median (IQR)	68 (58, 75)	62 (47, 71)	0.062	0.453	65 (52, 74)	69 (61, 72)	0.023
Female, n (%)	45 (47.9)	18 (45.0)	0.851	0.058	83.0 (48.6)	76.5 (51.2)	0.050
Endocarditis Site, n (%)			0.032	0.541			0.185
Native Valve	62 (66.0)	35 (87.5)			120.4 (70.6)	113.5 (75.9)	
Native Valve + Prosthesis	1 (1.1)	0 (0.0)			1.0 (0.6)	0.0 (0.0)	
Prosthesis	30 (31.9)	5 (12.5)			48.2 (28.3)	36.1 (24.1)	
Blood Culture, n (%)	86 (96.6)	40 (100.0)	0.552	0.264	162.6 (98.2)	149.6 (100.0)	0.192
Negative Blood Culture, n (%)	8 (9.2)	5 (12.5)	0.546	0.106	21.3 (13.0)	20.8 (13.9)	0.027
Staphylococcus Aureus, n (%)	26 (30.2)	11 (28.9)	1.000	0.028	36.4 (22.4)	32.3 (23.1)	0.017
Staphylococcus Non-Aureus, n (%)	16 (18.6)	1 (2.6)	0.021	0.537	23.8 (14.6)	16.5 (11.8)	0.082
Streptococcus, n (%)	16 (18.6)	15 (39.5)	0.023	0.472	50.3 (30.9)	46.5 (33.3)	0.050
Pseudomonas, n (%)	1 (1.2)	0 (0.0)	1.000	0.153	1.0 (0.6)	0.0 (0.0)	0.111
Enterococcus Faecalis, n (%)	8 (9.3)	5 (13.2)	0.535	0.122	14.7 (9.1)	12.1 (8.7)	0.014
Fungus, n (%)	1 (1.2)	0 (0.0)	1.000	0.153	1.0 (0.6)	0.0 (0.0)	0.111
Other Pathogen, n (%)	10 (11.6)	2 (5.3)	0.341	0.230	19.1 (11.7)	20.3 (14.5)	0.083
Hypertension, n (%)	65 (69.1)	19 (47.5)	0.020	0.450	110.6 (64.8)	96.9 (64.8)	0.001
Diabetes, n (%)	20 (21.3)	9 (22.5)	1.000	0.030	51.8 (30.4)	49.8 (33.3)	0.063
Obesity, n (%)	24 (25.5)	5 (12.5)	0.112	0.337	37.3 (21.8)	29.6 (19.8)	0.050
COPD, n (%)	12 (12.8)	4 (10.0)	0.777	0.087	18.9 (11.1)	14.0 (9.4)	0.057
EF, median (IQR)	60 (55, 65)	60 (55, 61)	0.627	0.082	60 (55, 61)	60 (55, 63)	0.015
Drug Addiction, n (%)	6 (6.4)	2 (5.0)	1.000	0.060	6.0 (3.5)	5.0 (3.4)	0.009
Redo, n (%)	39 (41.5)	6 (15.0)	0.003	0.616	66.0 (38.7)	51.7 (34.6)	0.086
Number Of Prior Valves, median (IQR)	0.0 (0.0, 1.0)	0.0 (0.0, 0.0)	0.008	0.390	0.0 (0.0, 1.0)	0.0 (0.0, 0.2)	0.050
Preoperative IABP, n (%)	1 (1.1)	0 (0.0)	1.000	0.147	1.0 (0.6)	0.0 (0.0)	0.109
Peripheral Artery Disease, n (%)	9 (9.6)	2 (5.0)	0.505	0.177	13.0 (7.6)	10.6 (7.1)	0.020
Malignancy, n (%)	7 (7.4)	4 (10.0)	0.732	0.091	12.9 (7.6)	11.7 (7.8)	0.009
Neurological Disease, n (%)	26 (27.7)	5 (12.5)	0.073	0.385	31.7 (18.6)	27.5 (18.4)	0.005
Unstable Angina, n (%)	3 (3.2)	1 (2.5)	1.000	0.042	3.0 (1.8)	1.0 (0.7)	0.100
Shock, n (%)	6 (6.4)	4 (10.0)	0.485	0.132	13.7 (8.0)	13.4 (9.0)	0.033
Heart Failure, n (%)	23 (24.5)	5 (12.5)	0.164	0.312	34.8 (20.4)	26.6 (17.8)	0.067
MI Within 90 Days, n (%)	1 (1.1)	0 (0.0)	1.000	0.147	1.0 (0.6)	0.0 (0.0)	0.109
Active Endocarditis, n (%)	76 (80.9)	27 (67.5)	0.118	0.309	123.3 (72.3)	105.0 (70.2)	0.045
Preoperative Intubation, n (%)	8 (8.5)	4 (10.0)	0.751	0.051	15.7 (9.2)	13.4 (9.0)	0.009
PAPs > 50 Mmhg, n (%)	10 (10.6)	0 (0.0)	0.033	0.488	10.0 (5.9)	0.0 (0.0)	0.353
Logistic Euroscore, median (IQR)	17.9 (8.1, 31.2)	7.9 (4.4, 20.1)	0.002	0.561	13.1(6.0, 27.8)	13.6 (6.3, 36.0)	0.096
Cirrhosis, n (%)	1 (1.1)	0 (0.0)	1.000	0.147	1.0 (0.6)	0.0 (0.0)	0.109
Chronic Kidney Disease (creatinine > 2 mg/dL), n (%)	12 (12.8)	5 (12.5)	1.000	0.008	20.9 (12.2)	18.2 (12.2)	0.002
Dialysis, n (%)	7 (7.4)	1 (2.5)	0.435	0.229	7.0 (4.1)	5.5 (3.7)	0.021
Pacemaker, n (%)	3 (3.2)	1 (2.5)	1.000	0.042	3.4 (2.0)	2.0 (1.4)	0.047

COPD: Chronic Obstructive Pulmonary Disease; EF: Ejection Fraction; IABP: Intra-Aortic Balloon Pump; IQR: Interquartile Range; PAPs: Pulmonary Artery Pressures.

**Table 2:** Operative characteristics.

	Before IPTW				After IPTW		
	Full	Mini	*p*-Value	SMD	Full	Mini	SMD
n	94	40			170.57	149.59	
Abscesses, n (%)	16 (17.0)	1 (2.5)	0.022	0.505	16.0 (9.4)	10.4 (6.9)	0.089
Vegetations, n (%)	75 (79.8)	38 (95.0)	0.036	0.471	150.8 (88.4)	139.5 (93.2)	0.168
Leaflet Perforation, n (%)	21 (22.3)	7 (17.5)	0.645	0.121	27.8 (16.3)	21.1 (14.1)	0.062
Prosthesis Detachment, n (%)	16 (17.0)	2 (5.0)	0.094	0.391	24.1 (14.2)	17.5 (11.7)	0.072
Duration Of IE (Days), median (IQR)	20 (13, 45)	26 (13, 46)	0.518	0.101	23 (11, 46)	25 (10, 47)	0.046
Mitral Valve, n (%)			<0.001	0.728			0.624
Repair	13 (13.8)	18 (45.0)			29.6 (17.3)	67.2 (44.9)	
Replace	81 (86.2)	22 (55.0)			141.0 (82.7)	82.4 (55.1)	
Type of Valve, n (%)			0.141	0.411			0.749
Biological	42/81 (51.9)	32/40 (71.4)			94.7 (55.5)	130.4 (87.2)	
Mechanical	39/81 (48.1)	6/40 (28.6)			75.9 (44.5)	19.1 (12.8)	
Cardiopulmonary Bypass Time, median (IQR)	99 (78, 118)	111 (81, 124)	0.242	0.196	103 (81, 115)	87 (70, 118)	0.074
Clamping time, median (IQR)	81 (63, 97)	86 (63, 102)	0.461	0.119	87 (65, 98)	71 (55, 107)	0.140

IE: Infective Endocarditis; IQR: Interquartile Range.

**Table 3:** Post-operative characteristics.

	Before IPTW			After IPTW		
	Full	Mini	*p*-Value	Full	Mini	Mini vs. Full, Coef (OR or β), 95% CI, *p*
n	94	40		170.57	149.59	
Intubation Hours, median (IQR)	11 (6, 25)	7 (4, 11)	0.004	9 (5, 16)	7 (4, 10)	−0.854 (−1.735; 0.052) *p* = 0.059
ICU Stay (Days), median (IQR)	4.0 (2.0, 7.0)	2.0 (2.0, 4.3)	0.024	3.0 (2.0, 7.0)	2.0 (2.0, 4.5)	−0.542 (−0.987; −0.094) *p* = 0.018
Sepsis, n (%)	10 (10.6)	1 (2.5)	0.173	27.7 (16.2)	1.0 (0.7)	0.032 (0.004–0.241) *p* = 0.001
Multi-organ Failure, n (%)	2 (2.1)	2 (5.0)	0.582	9.7 (5.7)	6.6 (4.4)	0.722 (0.262–1.987) *p* = 0.528
Complete Heart Block with Pacemaker, n (%)	8 (8.5)	1 (2.5)	0.279	13.6 (8.0)	1.2 (0.8)	0.091 (0.013–0.622) *p* = 0.014
Cardiac Arrest with VF, n (%)	0 (0.0)	0 (0.0)	NA	0.0 (0.0)	0.0 (0.0)	/
Atrial Fibrillation, n (%)	25 (26.6)	8 (20.0)	0.514	29.7 (17.4)	15.0 (10.0)	0.535 (0.273–1.047) *p* = 0.068
Stroke, n (%)	7 (7.4)	1 (2.5)	0.435	14.9 (8.7)	1.0 (0.7)	0.066 (0.009–0.506) *p* = 0.009
Acute Kidney Injury, n (%)	11 (11.7)	4 (10.0)	1.000	20.9 (12.2)	8.4 (5.6)	0.420 (0.181–0.973) *p* = 0.043
Continuous Venovenous Hemofiltration, n (%)	4 (4.3)	2 (5.0)	1.000	11.7 (6.9)	6.6 (4.4)	0.591 (0.222–1.572) *p* = 0.292
Bleeding Volume (mL), median (IQR)	450 (250, 638)	350 (163, 450)	0.033	350 (261, 600)	329 (150, 396)	−0.348 (−0.787; 0.088) *p* = 0.120
Reoperation for Bleeding, n (%)	8 (8.5)	2 (5.0)	0.723	21.0 (12.3)	8.8 (5.9)	0.415 (0.182–0.942) *p* = 0.036
Reoperation for Dehiscence, n (%)	1 (1.1)	0 (0.0)	1.000	1.0 (0.6)	0.0 (0.0)	/
Length of Stay, median (IQR)	8.00 (6.00, 12.00)	7.00 (5.00, 10.00)	0.101	8.00 (6.00, 12.00)	7.48 (6.00, 10.67)	−0.179 (−0.423; 0.065) *p* = 0.151
Death, n (%)	6 (6.4)	2 (5.0)	1.000	13.9 (8.1)	6.6 (4.4)	0.492 (0.190–1.276) *p* = 0.145

ICU: Intensive Care Unit; IQR: Interquartile Range; VF: Ventricular Fibrillation.

## Data Availability

The data presented in this study are available on request from the corresponding author. The data are not publicly available due to data protection directive 95/46/EC.
